# Design Methodology for Magnetic Field-Based Soft Tri-Axis Tactile Sensors

**DOI:** 10.3390/s16091356

**Published:** 2016-08-24

**Authors:** Hongbo Wang, Greg de Boer, Junwai Kow, Ali Alazmani, Mazdak Ghajari, Robert Hewson, Peter Culmer

**Affiliations:** 1 School of Mechanical Engineering, University of Leeds, Leeds LS2 9JT, UK; el11jwk@leeds.ac.uk (J.K.); A.Alazmani@leeds.ac.uk (A.A.); P.R.Culmer@leeds.ac.uk (P.C.); 2 Department of Aeronautics, Imperial College London, London SW7 2AZ, UK; g.de-boer@imperial.ac.uk (G.d.B.); r.hewson@imperial.ac.uk (R.H.); 3 Dyson School of Design Engineering, Imperial College London, London SW7 2AZ, UK; m.ghajari@imperial.ac.uk

**Keywords:** tactile sensors, soft sensing, force sensors, Hall effect sensor, magnetic field, hyperelastic elastomer, silicone rubber, moving least square, calibration, design methodology

## Abstract

Tactile sensors are essential if robots are to safely interact with the external world and to dexterously manipulate objects. Current tactile sensors have limitations restricting their use, notably being too fragile or having limited performance. Magnetic field-based soft tactile sensors offer a potential improvement, being durable, low cost, accurate and high bandwidth, but they are relatively undeveloped because of the complexities involved in design and calibration. This paper presents a general design methodology for magnetic field-based three-axis soft tactile sensors, enabling researchers to easily develop specific tactile sensors for a variety of applications. All aspects (design, fabrication, calibration and evaluation) of the development of tri-axis soft tactile sensors are presented and discussed. A moving least square approach is used to decouple and convert the magnetic field signal to force output to eliminate non-linearity and cross-talk effects. A case study of a tactile sensor prototype, MagOne, was developed. This achieved a resolution of 1.42 mN in normal force measurement (0.71 mN in shear force), good output repeatability and has a maximum hysteresis error of 3.4%. These results outperform comparable sensors reported previously, highlighting the efficacy of our methodology for sensor design.

## 1. Introduction

The integration of tactile sensors into robotic systems is essential if such robots are to interact safely with the external environment and to dexterously manipulate objects [1,2,3]. Despite two decades of rapid development, tactile sensing technology remains relatively un-developed for widespread use because it must have both high compliance and high performance (like the human fingertip) and needs to be durable to survive the physical interaction with an unexpected world [4]. Current tactile sensors have limitations restricting their application, notably being too fragile for repeated contact/impact and wear or exhibiting poor performance. Furthermore, they are typically expensive and difficult to integrate into the application systems. Thus, there is a demand for low-cost, durable, accurate, deformable, customizable, tri-axial tactile sensing technology and the associated techniques required to design, optimize and fabricate these systems.

Over the past few decades, research into deformable/soft tactile sensing systems has rapidly accelerated, spanning a broad range of target applications [5]. Existing systems employ technologies including MEMS pressure sensors (TakkTile [6]), optical systems (TacTip [7], OptoForce [8]), conductive liquid (BioTac [9]), soft capacitive/resistive sensor [10,11,12] and magnetic field-based sensors [13,14]. Despite their success, these systems have significant limitations. TakkTile measures only normal force with limited bandwidth. Optical systems usually have high spatial resolution, but are power-hungry, bulky, have low force sensitivity, are low speed and require complex computation. BioTac is a commercialised multi-modal system (including low frequency force, vibration and temperature sensing) for robotic hands, providing force, contact area and temperature information. However, the force information from BioTac is limited in accuracy and resolution and requires a complicated signal processing unit. Other modalities have been explored. Soft capacitive sensor/resistive sensors are typically restricted to normal force measurement. Three-axis force measurement using capacitive sensors is possible, but requires a complicated fabrication procedure [10] and has a limited compliance when compared to the other sensor modalities listed above. 

More recently, magnetic field-based tactile sensors have shown potential to achieve both high compliance and high performance at a reasonable cost [14]. The idea of using magnets and magnetic sensors for soft tactile sensing was proposed by Clark [13] in 1988, but the system was limited by the magnetic field sensing technology at that time. With the substantial progress in magnetic field sensing technology (particularly Hall effect sensors) and the demand of low-cost, accurate, deformable tactile sensors, some tactile sensing systems using magnetic sensors were developed and integrated into robotic applications. By using a permanent magnet and a Hall sensor, a single-axis deformable tactile sensor was developed to measure the surface normal force for robotic hands and used for the classification of grasped objects in a real robotic system [15]. This highlighted that this technology has the potential to deliver low-cost, robust, low hysteresis, high sensitivity and repeatable sensors. In 2009 [16], a low-power magnetic-type tactile sensor was developed to measure three-axis force by using a two-dimensional array of inductors; however, this sensor was not capable of measuring static and slowly varying forces. A three-axis tactile sensor using four Hall sensors and a magnet with a soft spherical dome was developed [17], and the contact behaviours of the soft body were comprehensively analysed, but the sensor has limited accuracy and is difficult to miniaturize. The use of 3D Hall sensors for tactile sensing was first proposed in 2013 [14] and used to measure normal force. A tactile sensor using three Hall sensors [18] was proposed for biomedical applications in 2015; the sensor was developed to measure three-axis force, tip displacement and vibration; however, the calibration of the sensor was not fully investigated, and the evaluation was limited to normal force results. Most recently, a more mature test of the characterization of a three-axis soft tactile sensor using an integrated 3D Hall sensor and a block magnet [19] was published. Here, the sensor’s thermal drift was tested and compensated using pre-determined coefficients. The force output was derived from the magnetic field using quadratic regression. However, the results showed significant error (particularly in normal force), largely as a result of the crosstalk effect between the three magnetic field components, which were not decoupled. It is clear that while the field of soft tactile sensing is developing rapidly, it is relatively immature. In this area, using a magnetic field sensing modality has seen increasing popularity due to the availability of low-cost, multi-axis MEMS Hall effect sensors capable of high precision measurements. However, it is also evident that aspects of the design, fabrication and operation are challenging and relate directly to sensor system performance. The design requires the consideration of interrelated components, including the soft body, Hall effect sensor and magnet. Fabrication methods are typically manual and susceptible to variability in quality. The operation requires characterisation and calibration of the non-linear relationship between the applied force and the measured magnetic field in multiple degrees of freedom. Characterisation from first-principles derivation may induce errors due to discrepancies between the idealised and actual sensors (e.g., [19]) occurring during fabrication. These challenges may therefore act as a barrier to more widespread use of this promising technology.

In this manuscript, we present a general design methodology for magnetic field-based three-axis soft tactile sensors, enabling researchers to more easily and rigorously develop and integrate their own tactile sensors into a variety of applications. The design, fabrication, characterisation and performance evaluation were fully investigated and discussed to directly address the complexities and uncertainties of magnetic field-based tactile sensors and, thus, facilitate improved performance, reliability and robustness. Furthermore, a multi-variable polynomial phenomenological model was introduced to express the relationship between the force and the magnetic field in which the coefficients are calculated by the moving least square (MLS) method. This facilitates an accurate tri-axis force output with a minimised cross-talk effect. Section 2 introduces the general working principles of magnetic tri-axis soft tactile sensors, then proceeds to investigate their design, fabrication and calibration. In Section 3, the design methodology is demonstrated using a case study on the design and evaluation of a sensor prototype (MagOne). Section 4 presents the results from the evaluation of MagOne. These are discussed in Section 5, providing context for a more general consideration of the advantages and disadvantages of magnetic field-based tri-axis tactile sensors and the potential for future developments. 

## 2. Methodology 

### 2.1. Sensor Concept

**Figure 1:**
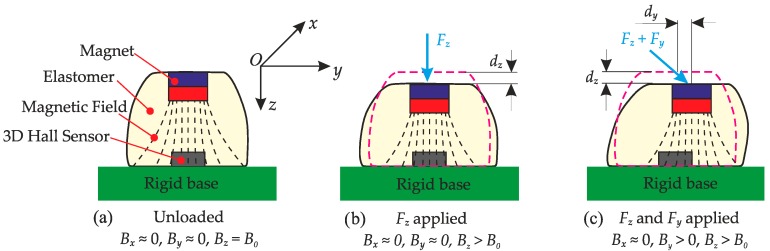
The concept of magnetic field-based three-axis soft tactile sensor: (**a**) Schematic of the tactile sensor (unloaded); (**b**) Tactile sensor (*F_z_* applied); (**c**) Tactile sensor (*F_z_* and *F_y_* applied).

As originally conceived of in [14], the tactile sensor comprises a 3D Hall sensor, a deformable elastomer and an embedded magnet. As shown in Figure 1, when the external force (normal and/or shear) is applied to the surface of the elastomer, the magnet will be displaced. By measuring the magnetic field vector through the Hall sensor, the three-axis displacement of the magnet can be obtained. The force applied to the elastomer can then be extracted based on the elastomer’s mechanical behaviour. 

The design of the tactile sensor can be classified into two separate parts. One is to design a magnetic field-based tri-axial displacement sensor, which can measure the tri-axis movement of the magnet. The other is to design the deformable probe, which undergoes a repeatable and predictable deformation subject to external surface loading. The relationship between these quantities is:

(1) $$\{\begin{array}{c} D=f_{1}\left(B\right)\\ F=f_{2}\left(D\right) \end{array}$$

where ***D*** is the vector describing the magnet’s movement, ***B*** is the magnetic field vector, ***F*** is the vector of the applied force and $f_{1}$ and $f_{2}$ are functions that define their relationships. Thus, the external force can be obtained from the magnetic field:

(2) $$F=f_{1}\left[f_{2}\left(B\right)\right]=g\left(B\right)$$

where *g* is function that represents the relationship between ***F*** and ***B***. The key point here is to determine the correlation between the magnetic field vector and the force vector. 

Theoretical analysis and the finite element method (FEM) were used to investigate the characteristics of the magnetic field gradient of the magnet and the mechanical behaviour of the elastomer. As the magnetic position sensor and the hyperelastic transfer body (elastomer) operate independently, they are analysed separately in Section 2.2 and Section 2.3. Then, the integration of these elements and the compound performance of the soft tactile sensor are discussed in Section 2.4.

### 2.2. Magnet-Based Position Sensing

#### 2.2.1. Magnetic Field Characterisation

**Figure 2:**
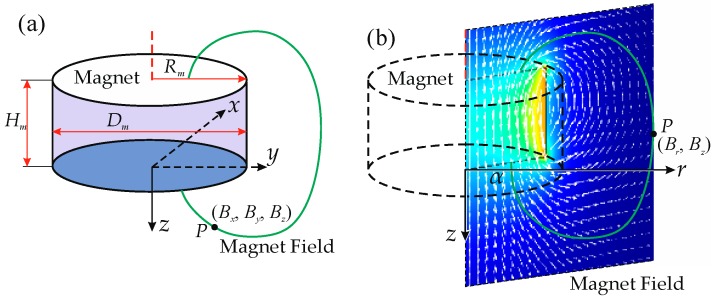
(**a**) A cylindrical permanent magnet and its magnetic field at point *P* in the Cartesian coordinate system; (**b**) The magnetic field vectors of the magnet in one axisymmetric plane and the magnetic field at point *P* in the cylindrical coordinate system.

As described by Schott [20], when the magnet is displaced, the relative direction and distance between the magnet and the sensor location (observed point) change, which is equivalent to moving the sensor location in the magnetic field of a magnet with a fixed position. Thus, we can simply investigate the magnetic field from the magnet to obtain the behaviour of the magnetic position sensor. Here, an axis-magnetized cylindrical permanent magnet is used as a source with an idealized 3D magnetic sensor to measure the three axis magnetic field density at point *P*, as shown in Figure 2. As the magnet and its magnetic field are axisymmetric, the 3D model can be described from the rotated 2D map. Figure 2b shows the magnetic field vector in one axisymmetric plane. The magnetic field at point *P* and the coordinates of *P* in two models meet the following equations [20]:

(3) $$\{\begin{array}{c} r=\sqrt{x^{2}+y^{2}}\\ B_{r}=\sqrt{B_{x}^{2}+B_{y}^{2}}\\ \frac{x}{y}=\frac{B_{x}}{B_{y}} \end{array}$$

Then, the coordinates of point *P* can be calculated in the 2D model by the following equation:

(4) $$\{\begin{array}{c} x=r\cdot \frac{B_{x}}{B_{r}}\\ y=r\cdot \frac{B_{y}}{B_{r}} \end{array}$$

Thus, the relationship between (*B_z_*, *B_r_*) and (*z*, *r*) is sufficient to obtain the three-axis displacement ***D*** (*x*, *y*, *z*) from the three-axis magnet field ***B*** (*B_x_*, *B_y_*, *B_z_*).

**Figure 3:**
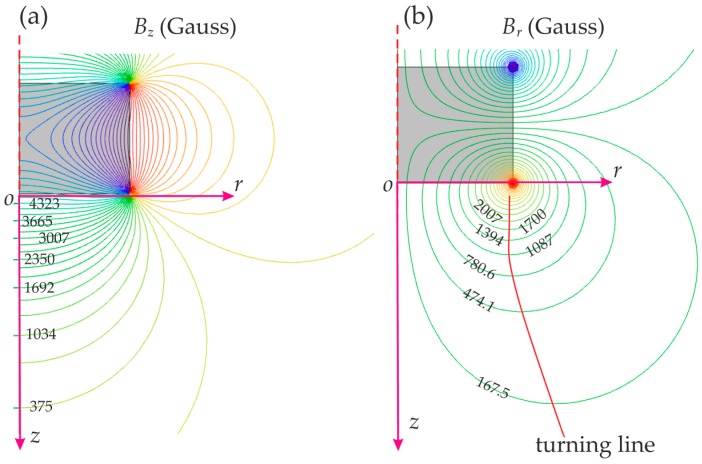
Magnetic field of a cylinder magnet in the *z*-*r* plane (*H_m_* = 0.5 *R_m_*): (**a**) *B_z_* contour; (**b**) *B_r_* contour.

Figure 3 shows the contour figures of the magnetic field density *B_r_* and *B_z_* of a cylinder magnet (radius: *R_m_*; height: *H_m_* *=* 0.5 *R_m_*) in the *z*-*r* plane, which implies two features: (1) non-linearity: both *B_z_* and *B_r_* in the *z*-*r* plane are not varying linearly with spatial location; (2) crosstalk effect: the magnetic field in the *z* axis (*B_z_*) changes with both *z* and *r*, the same as *B_r_*. These features make correlation between the displacement ***D*** and the magnetic field ***B*** non-trivial and difficult to solve analytically. The sensitivity of the magnetic position sensor ***S_B_*** is defined as:

(5) $$S_{B}=\frac{dB}{dD}$$

Figure 3 also indicates that the d*B_z_*/d*z* and d*B_r_*/d*r* components are dominant relative to d*B_r_*/d*z* and d*B_z_*/d*r*, when *r* < *R_m_*; while d*B_r_*/d*z* and d*B_z_*/d*r* cannot be ignored, as they still can significantly influence the position results. In addition, *B_r_* increases with *r* from zero to its maximum value (on the red solid line), then decreases to zero as *r* increases further. To avoid multiple results when determining *r* from the magnetic field, the movement of the magnet should not exceed this line (approximate to *r* = *R_m_*).

#### 2.2.2. Dimension Effect

Precisely calculating the magnetic field of a permanent magnet requires numerical computation with multiple integration and accurate parameters. For the axis magnetized cylindrical magnet in Figure 2, the magnetic field along its axis can be derived [21]:

(6) $$B\left(z\right)=\frac{\mu_{0}M}{2}\left(\frac{z+H_{m}}{\sqrt{{\left(z+H_{m}\right)}^{2}+{\left(D_{m}/2\right)}^{2}}}-\frac{z}{\sqrt{z^{2}+{\left(D_{m}/2\right)}^{2}}}\right)$$

where *μ_0_* is the relative magnetic permeability, *M* is the magnetization of the magnet and *z* is distance from a pole face on the symmetry axis. Equation (6) implies that for small magnets, the magnetic field strength will drop quickly and become too weak to be distinguished from the environmental magnetic field (limiting the position measuring range), but will have a much larger spatial gradient (sensitivity of position measurement), with the opposite true for larger magnets.

**Figure 4:**
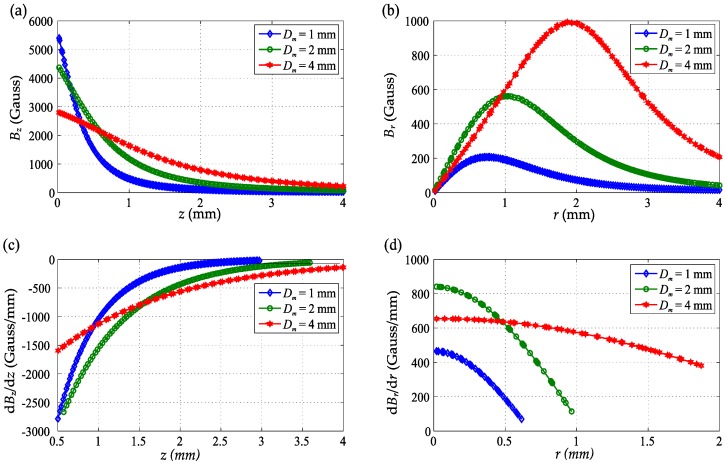
The magnetic field comparison of different sizes of magnets (*H_m_*= 1 mm, *D_m_*= 1, 2, 4 mm): (**a**) *B_z_* with *z* at *r* = 0 mm; (**b**) *B_r_* with *r* at *z* = 1 mm; (**c**) *B_z_* gradient in *z* axis; (**d**) *B_r_* gradient in *r* axis.

To quantitatively investigate the dimension effect of the magnet’s magnetic field, the magnetic fields in the *z*-*r* plane of different sizes of magnets were simulated using a 2D axisymmetric finite element model [22] (COMSOL Multiphysics 5.1, COMSOL Inc., Stockholm, Sweden). Figure 4 shows the magnetic field distribution and its spatial gradient in both the *z* and *r* axis of magnets for three different sizes. The magnetic field of a larger magnet has a much smaller *z* axis gradient of the $B_{z}$ field component ($dB_{z}/dz$), but a larger strong magnetic field area (effective for position sensing), while the magnetic field gradient of the $B_{r}$ component in the *r* axis (d*B_r_*/d*r*) is larger. As shown in Figure 4, both the magnetic field gradient and the magnetic field density decrease rapidly when distance *z* increases. Thus, the initial position (maximum distance between the magnet and the sensor location *P*) should be chosen to maintain a sufficient level of sensitivity, while the maximum displaced position (minimal distance) is limited by the maximum magnetic field density to avoid saturating the sensor.

#### 2.2.3. Magnetic Sensor

There are a range of magnetic field sensors, using a variety of technologies from Hall effect, giant magnetoresistance (GMR), anisotropic magnetoresistance (AMR) to MEMS Lorentz force, induction coil, flux gate effect and other magnetic phenomena, which provide a measuring resolution from pT to mT (1 mT = 10 Gauss) with a bandwidth from DC to MHz. A detailed introduction to these sensors and their performance can be found in [22,23]. Hall sensors [24] are widely used in industrial applications (e.g., position sensing, proximity sensing, current sensing, etc.), as they are inexpensive, small and easy to interface with data acquisition systems. Hall sensors have a large measuring range up to 1 T (10,000 Gauss) to span the strong magnetic field of permanent magnets. Hall sensors can suffer from issues, such as drift, poor temperature stability, high power consumption and single-axis operation. With the advancements in chopper stabilization, low-power circuitry and the latest 3D Hall technology, state-of-the-art Hall sensors [25] enable the measurement of magnetic fields in three axes from a single chip, typically with a small size (3 mm × 3 mm × 0.8 mm), low-power consumption (100 μW to 10 mW), digital output (via I2C or SPI bus) and an integrated temperature sensor for thermal drift compensation [26]. Recent examples of commercially-available sensors include the MLX90393 (Melexis, Ieper, Belgium) [27], TLV493D-A1B6 (Infineon, Neubiberg, Germany) [28], and AS54XX (ams, Premstaetten, Austria) [29].

As soft tactile sensors work in a variety of locations, the Hall sensor will measure the total magnetic field present in the environment, not only from the magnet, but also from other sources (e.g., geomagnetism, electromagnetic devices, other nearby magnetic materials). Therefore, the magnetic sensor requires a resolution that is able to discriminate the variation of the environmental magnetic field and has a measurement range as large as possible to avoid saturation when the magnet is compressed toward the sensor. For example, if the variation of the stray magnetic field in the environment is around 0.1 Gauss, then a resolution of 0.1 Gauss would be appropriate for the tactile sensors. A higher resolution of magnetic sensors would not necessarily improve the tactile sensing resolution as the system cannot discriminate whether the magnetic field variation is caused by the movement of the magnet or the variation of another source in the environment; the exception being the addition of another magnetic sensor to provide the reference signal of the environmental magnetic field, as discussed in Section 5.

### 2.3. Elastomer Design

The second part is designing the elastomer. The elastomer is the physical body that supports the magnet. It directly interacts with the objects and transfers the applied force to the displacement of the magnet. The sensitivity of the force measurement depends on the stiffness of the elastomer, ***K****_e_*, defined as:

(7) $$K_{e}=\frac{dF}{dD}$$

The magnet embedded in a softer elastomer will be displaced more under a certain force than in a stiffer elastomer, which results in higher sensitivity.

For the general sensing probe shown in Figure 1a, the normal and shear force with applied displacement can be estimated using Euler–Bernoulli beam theory [30] by simplifying the elastomer as a cantilever beam with a diameter of *D_e_* and a height of *H_e_*. Thus, the normal and shear stiffness of the simplified cylinder elastomer can be approximated [31]. However, as the materials for elastomers are typically hyperelastic and undergo large deformations in this application, the assumptions of linear elastic theory are no longer valid, and the resultant predictions must be treated with caution.

**Figure 5:**
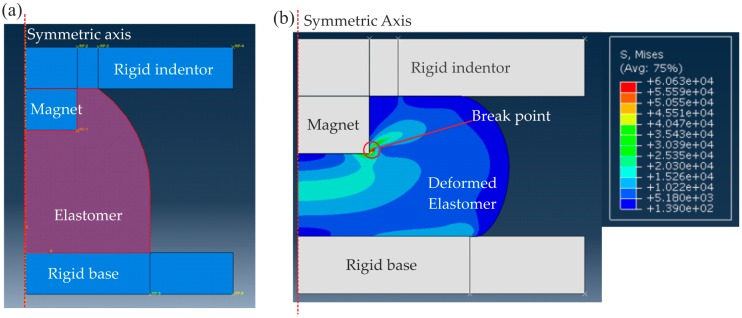
(**a**) 2D symmetric FE model of the elastomer when it is fixed on a rigid base and indented by a rigid flat surface; (**b**) The von Mises stress distribution in the elastomer when it is indented by 3 mm.

For an improved model of the sensor mechanics, which was needed to calculate the correlation between force and the displacement of the magnet, a 2D axisymmetric finite element model (Figure 5a) was built (ABAQUS, Dassault Systèmes, Yvelines, France). An incompressible neo-Hookean hyperelastic material model [32] was used for the elastomer. Rigid bodies were used to simulate the magnet, base and indentation surfaces, and the contact mechanics was specified in the regions where the rigid surfaces interact with the deformable rubber-like silicone during indentation (frictionless, hard contact algorithm in ABAQUS).

**Figure 6:**
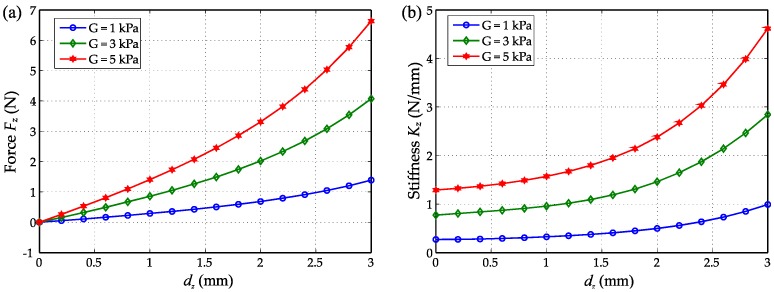
(**a**) The indenting force *F_z_* with the indentation depth *d_z_* for different materials (Shear modulus G = 1, 3, 5 kPa); (**b**) The compressive stiffness of the elastomer with the indentation depth for different materials.

The stress in the silicone is shown in Figure 5b when the magnet is displaced by 3 mm, which shows stress concentration on the edge of magnet and the rigid base. The maximum stress allowed is limited by the break strength of the elastomer material. Thus, for a selected material, the maximum deformation (magnet displacement) $d_{z\_max}$ is limited by the maximum stress encountered by the elastomer. A range of shear modulus (G) between 1 and 5 kPa was considered to obtain the force response during indentation. Figure 6a demonstrates that force increases nonlinearly with indentation depth *d_z_* because of the hyperelastic material model used. The stiffer material (G = 5 kPa) deviates further from a linear response than the soft material (G = 1 kPa). As can be seen in Figure 6b, which shows that the stiffness increases with the indentation depth.

In this elastomer design, the magnet was positioned close to the top surface of the elastomer instead of embedding the magnet in the middle of the elastomer (in most previous studies), to maximise the dynamic response of the sensor. The magnet will be displaced immediately when force is applied to the elastomer surface with minimal lag due to the compression of the silicon above; thus, detection of the force will be responsive. This design enables the sensor to achieve the best bandwidth. In addition, most silicone-like rubber materials have a relatively strong hysteresis and creep effect [33] because of their viscoelasticity. These effects will reduce the accuracy of tactile sensors if they are not taken into account in the sensor design. Alternative elastomer structures, geometries or advanced soft materials could be used to reduce these effects when stability and low-hysteresis are crucial factors for the sensor.

### 2.4. Analysis of Sensor Performance

**Figure 7:**
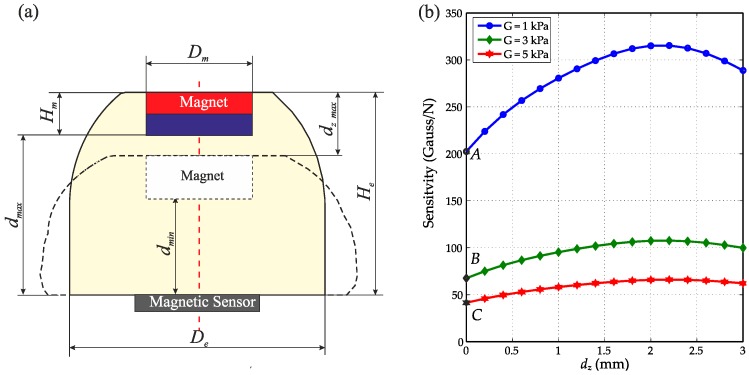
(**a**) The design parameters of the tactile sensor; (**b**) The sensitivity of the tactile sensor with different material properties (shear modulus G = 1, 3, 5 kPa). Points A–C indicate the point of lowest sensitivity for each material.

As shown in Figure 7a, the design parameters of the tactile sensor include the diameter and height of the magnet (*D_m_* and *H_m_*), the diameter and height of the elastomer (*D_e_* and *H_e_*) and the maximum deformation $d_{z\_max}$, which has the relationship:

(8) $$d_{z\_max}=d_{max}-d_{min}$$

where $d_{max}$ and $d_{min}$ are the maximum and the minimum distance between the magnet surface and the Hall sensor. Based on the analysis and discussion in the two above sections, these parameters should meet the following constraints:

(9) $$\{\begin{array}{c} d_{r\_max}\leq \frac{D_{m}}{2}=R_{m}\\ B\left(d_{min}\right)\leq B_{sat}\\ \frac{dB}{dz}{|}_{z=d_{max}}\geq {\left(S_{B}\right)}_{min}\\ F_{z\_range}=F\left(d_{z\_max}\right) \end{array}$$

where *B_sat_* is the saturation magnetic field of the sensor and *F_z_range_* is the force measuring range in the z axis. According to Equations (5) and (7), the sensitivity of the tactile sensor ***S_F_*** can be expressed as:

(10) $$S_{F}=\frac{dB}{dF}=\frac{dB}{dD}\cdot \frac{dD}{dF}=\frac{S_{B}}{K_{e}}$$

Thus, we can either use ***S_B_*** and ***K****_e_* to calculated the force sensitivity or directly derive the correlation between force and magnetic field. However, due to the strong crosstalk-effect and nonlinear relationship between these parameters, determining the correlation is challenging, which is discussed in Section 2.5. When the magnetic field noise of the environments can be measured and indicated as *N_B_*, the resolution of the tactile sensor ***R*** can be calculated as:

(11) $$R=\frac{N_{B}}{S_{F}}$$

By using the simulation results of the elastomer shown in Figure 6b and the calculated magnetic gradient of a magnet with a size of 5 mm × 2 mm ($D_{m}\times H_{m}$), the sensitivity of the tactile sensor with three different materials was calculated. As shown in Figure 7b, the tactile sensor has its minimum sensitivity at zero deformation (Points A, B and C). Decreasing (increasing) the shear modulus of the elastomer materials can proportionately increase (or decrease) the sensitivity of the tactile sensor. While there is a compromise between sensitivity and maximum force, softer materials result in higher sensitivity, but a smaller measuring range (maximum force). As ultra-soft material might be too fragile for some applications, an alternative method is to change the geometry (size, height/diameter, etc.) or structure (e.g., using an air chamber inside the elastomer) of the elastomer to enhance the sensitivity and resolution. The worst-case resolution can be found using Equation (11) with the lowest sensitivity for that material (see Figure 7) and an estimation of the root mean square (RMS) magnetic field noise in the environment. In our laboratory, we found N_B_ = 0.1 Gauss, giving a normal force resolution of 0.49 mN, 1.47 mN and 2.41 mN for the three elastomer materials shown in Figure 7b at Points A, B and C, respectively.

### 2.5. Sensor Calibration

As analysed in the above sections, the correlation between the magnetic field and the force applied is very complicated as they are highly non-linear and have a strong cross-talk effect. Here, a multiple-parameter polynomial is introduced to express the correlation between the magnetic field and the applied force:

(12) $$\{\begin{array}{c} F_{z}=\overset{n}{\underset{k=0}{\sum }}\overset{k}{\underset{i=0}{\sum }}C_{zj}\cdot B_{z}^{i}\cdot B_{r}^{k-i}\\ F_{r}=\overset{n}{\underset{k=0}{\sum }}\overset{k}{\underset{i=0}{\sum }}C_{rj}\cdot B_{z}^{i}\cdot B_{r}^{k-i} \end{array}$$

where *F_z_* and *F_r_* are normal and shear force, *C_zj_* and *C_rj_* are the coefficients of the polynomials for *F_z_* and *F_r_*, *j* is the index of the coefficient and *n* is the order of the polynomial, which can be increased or decreased to meet the required accuracy. The moving least squares (MLS) method [34] was used to calculate the best fitting polynomials. When the order of the polynomial is selected as 3, the equation to calculate the normal and shear force can be expressed as:

(13) $$\left[\begin{array}{c} F_{r}\\ F_{z} \end{array}\right]=\left[\begin{array}{c} C_{r1}\;C_{r2}\ldots \;C_{r9}\;C_{r10}\\ C_{z1}\;C_{z2}\ldots \;C_{z9}\;C_{z10} \end{array}\right]\cdot {\left[1\;B_{z}\;B_{r}\;B_{z}^{2}\;B_{z}B_{r}\;B_{r}^{2}\;B_{z}^{3}\;B_{z}^{2}B_{r}\;B_{z}B_{r}^{2}\;B_{r}^{3}\right]}^{T}$$

In order to obtain these coefficients, a 2D scanning process allows the collection of a dataset across a range of applied forces and their corresponding magnetic field values. This dataset should include at least 10 pairs (across all axes) of magnetic field and force and should cover a wide force range to ensure the whole measuring range is calibrated. When there are more points in the dataset than the number of the coefficients, these coefficients determine the least square error of the over-determined system. By performing the MLS regression with the 2D scanning dataset, the coefficients in Equation (13) can be obtained, then the three-axis force can be calculated from three magnetic field signals. According to Equations (3) and (4), shear force in the *x* and *y* axes can then be calculated by:

(14) $$\{\begin{array}{c} F_{x}=\frac{B_{x}}{\sqrt{B_{x}^{2}+B_{y}^{2}}}\cdot F_{r}\\ F_{y}=\frac{B_{y}}{\sqrt{B_{x}^{2}+B_{y}^{2}}}\cdot F_{r} \end{array}$$

Thus, the three-axis force can be calculated from the three-axis magnetic field signal. Using the same method, the displacement of the magnet ***D*** also can be calculated, providing an output, which could be used to detect the slip between the sensor tip and the object.

### 2.6. Design Guidelines

From the analyses presented in this section, a series of design guidelines for a magnetic field-based tactile sensor can be summarised:

(1) Magnetic source: choose the magnet size, such that the radius of the magnet is larger than the maximum tangential displacement of the magnet. The height of the magnet should be as small as possible to reduce the weight, but should be large enough to maintain a strong magnetic field in the *z* axis. A ratio of 0.2~0.5 between the magnet height and diameter is appropriate for most situations.
(2) Sensitivity and range: The maximum distance between the magnet and the magnetic sensor *d_max_* should be small enough so that it will meet the minimum magnetic field gradient (*S_B_*)*_min_* requirement. Generally, a maximum distance *d_max_* between 1- and 2-times of the magnet diameter (*D_m_*) is appropriate. Based on the saturation magnetic field of the magnetic sensor, the minimum distance should be selected to avoid saturating the sensor and, also, should fit the maximum deformation requirement.
(3) Elastomer geometry: Once *d_max_* and the magnet height *H_m_* are defined, the height of the elastomer *H_e_* is determined. The diameter of the elastomer alters both the compressive stiffness and the shear stiffness. The fabrication and application limitations should also be considered when choosing the appropriate diameter.
(4) Material properties: Changing the elastomer material can change the stiffness of the elastomer, resulting in decreasing (or increasing) the sensitivities of both normal and shear force measurement. The stiffness of the elastomer defines the range of force measurement, as well.

## 3. Design Case Study: The MagOne Tactile Sensor

In this section, we present a case-study concerning the design, fabrication, calibration and validation of a magnetic field-based soft tri-axial tactile sensor “MagOne”, using the methodology presented in Section 2.

### 3.1. Design

The MagOne sensor was developed to investigate the performance that can be obtained from a finger-tip-sized sensor using the latest commercially-available 3D Hall effect technology. We therefore used the guidelines presented in Section 2.6 to shape the design of this system.

**Figure 8:**
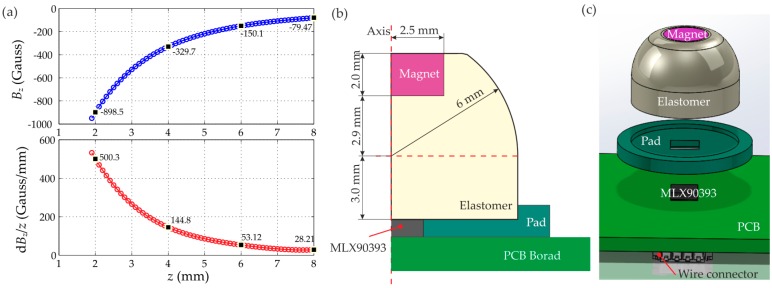
(**a**) Magnetic field Bz and its gradient along the z axis; (**b**) A cross-section of the MagOne sensor with dimensions; (**c**) Design of the MagOne prototype.

(1) Magnetic source: To achieve a wide measurement range (i.e., deformation), an axis-magnetized cylindrical magnet (N35 neodymium, First4Magnets) with a diameter of 5 mm and a height of 2 mm was selected for MagOne, which will allow a deformation approximately the diameter of the magnet.
(2) Sensitivity and range: The magnetic field density and gradient along the *z* axis for the selected magnet are shown in Figure 8a. Based on this, the maximum distance between the magnet and sensor was selected as 6 mm, where the magnetic field density is 150 Gauss, and the gradient is 53 Gauss/mm. A compact 3D Hall effect sensor, MLX90393 (3 mm × 3 mm × 0.8 mm, QFN-16 package), with digital output (via I2C bus) was selected to measure the three-axis magnetic field. The magnetic sensor has a configurable measuring range up to ±962 Gauss in the *z* axis, with a 16-bit ADC [27]. The sensor will saturate when the distance is less than 1.9 mm (*B_z_* = 950 Gauss); therefore, the minimum distance *d_min_* can be determined as 2 mm, and the maximum deformation *d_z_max_* is 4 mm (Equation (8)), where the magnetic gradient is as high as 500 Gauss/mm. Considering the large stress concentration on the edge of the magnet, the maximum deformation is limited to 3 mm, where the magnetic gradient is 265 Gauss/mm.
(3) Elastomer geometry: As shown in Figure 8b, an elastomer with a cylinder base and hemisphere tip was used as the transfer medium, and the magnet was embedded on the top of the elastomer. Based on the distance determined above, the elastomer has a total height of 7.9 mm, and a diameter of 12 mm was selected here to make sure that the elastomer has a comparable shear stiffness to compressive stiffness and that the sensor probe will not bend over.
(4) Material properties: Silicone with a shore hardness of 00-30 was selected for the elastomer, which has a shear modulus of 3.06 kPa. By using the finite element model presented in Figure 5, the stiffness of the sensor can be determined for specific load conditions, then the sensitivity and the resolution of the tactile sensor can be estimated.

**Figure 9:**
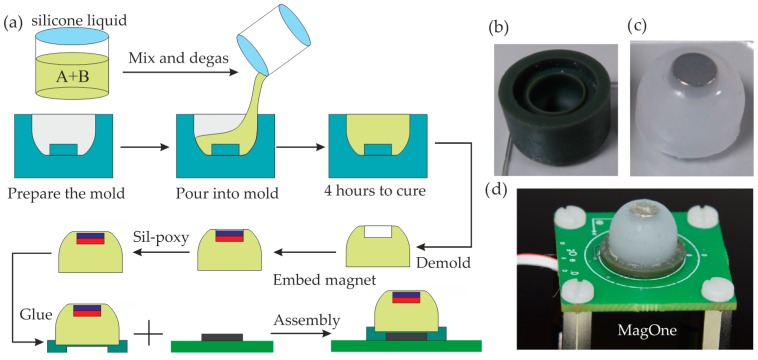
(**a**) Schematic of the fabrication process; (**b**) Photograph of the mould; (**c**) Photograph of the fabricated elastomer; (**d**) Photograph of the MagOne prototype.

Figure 9b,c show the dimension and the full design of the MagOne prototype. The elastomer with magnet embedded can be glued on a rigid disk-shaped pad with a square hole, which can be perfectly fit into the sensor chip on the printed circuit board (PCB).

### 3.2. Fabrication

The fabrication of MagOne comprises three steps: (1) PCB fabrication and electronics assembly; (2) fabrication of the elastomer with the magnet embedded; (3) assembly of the prototype. Firstly, the PCB was designed to mount the 3D Hall sensor MLX90393 on the centre of a 30 mm × 30 mm PCB, and a compact four-way connector was used for power and communication (via the I2C protocol) with the sensor chip from an external controller (myRIO-1900, National Instruments, Austin, TX, USA). The PCB was fabricated commercially, then the elastomer was fabricated using a silicone casting process according to the following protocol:

(1) An inverse mould was designed and then printed using a high-resolution 3D printer (Perfactory, EnvisionTEC, Gladbeck, Germany), shown in Figure 9b. Prior to casting, the mould was cleaned and treated with silicone release agent to prevent mould adhesion.
(2) The silicone (Ecoflex 00-30, Smooth-On Inc., Macungie, PA, USA) was prepared at 1:1 (Part A:Part B) by weight, then degassed through a vacuum pump.
(3) The degassed silicone liquid was poured slowly into the mould and left at room temperature for 4 h until fully cured before removal.
(4) The magnet is embedded into the hole in the silicone body and secured using a small volume of Sil-poxy (silicone adhesives, Smooth-On Inc.) to ensure that the magnet is fully encapsulated and integrated into the silicone elastomer body. The fabricated elastomer with magnet embedded is shown in Figure 9c.

Finally, the elastomer was glued on the mounting pad with Sil-poxy, then assembled on the PCB. A recess in the pad centres the magnetic sensor chip on the elastomer and embedded magnet. Figure 9d shows the photographs of the fabricated MagOne prototypes. A real-time LabView program (myRIO-1900) was developed to communicate with the Hall sensor via the I2C bus.

### 3.3. Calibration and Test Platform

**Figure 10:**
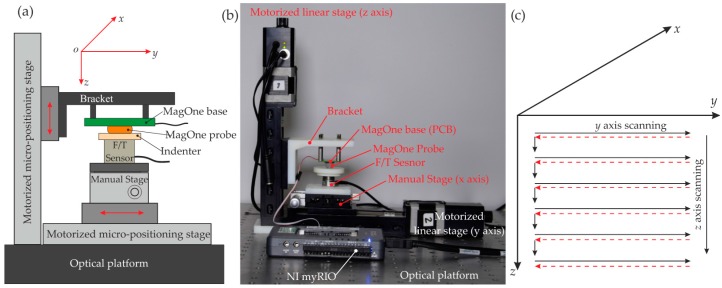
Test platform and calibration: (**a**) The schematic of the test platform; (**b**) A photograph of the test platform; (**c**) 2D scanning track for sensor calibration.

To obtain the correlation between the magnetic field output signal and the applied force and to evaluate the performance of the sensor, a custom test platform was developed. As shown in Figure 10, the platform comprises micro-positioning stages, a force/torque (F/T) sensor, the MagOne sensor and a holding bracket. They were assembled and mounted on an optical platform to minimise background noise from vibration during testing, as shown in Figure 10b. The micro-positioning system uses two motorized linear stages (T-LSR75B, Zaber Technologies Inc., Vancouver, BC, Canada) and one manual stage. One of the motorized stages moved the MagOne prototype in the *z* axis; the other motorized stage moved the F/T sensor and the indenting surface in the *y* axis; and the manual positioning stage was used to adjust the x position of the F/T sensor. The motorized linear stage has a minimum step of 0.5 μm, a travel range of 75 mm and repeatability of 2.5 μm. The F/T sensor (Nano17-E, ATI Industrial Automation, Apex, NC, USA) has a measuring range of ±35 N in the *z* axis (±25 N in *x*/*y* axis), with a resolution of 6.25 mN (in the *x*, *y* and *z* axis). A program (NI LabVIEW) was developed to control the movement of the motorized stages, to acquire data from the F/T and MagOne sensors and to record data into a measurement file. To obtain the reference force (from the F/T sensor) and the corresponding magnetic field across the measurement range, a 2D scanning process was performed along the track shown in Figure 10c.

## 4. Experimental Results

### 4.1. Sensor Calibration

**Figure 11:**
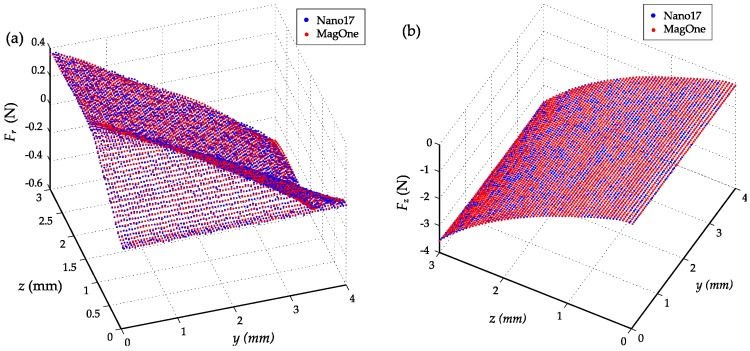
Comparison of the calibrated force output (red circle) from MagOne and the reference force from the F/T sensor (blue circle) during the *y*-*z* 2D scanning process: (**a**) Shear force *F_r_* in the *z*-*y* plane; (**b**) Normal force *F_z_* in the *z*-*y* plane.

**Figure 12:**
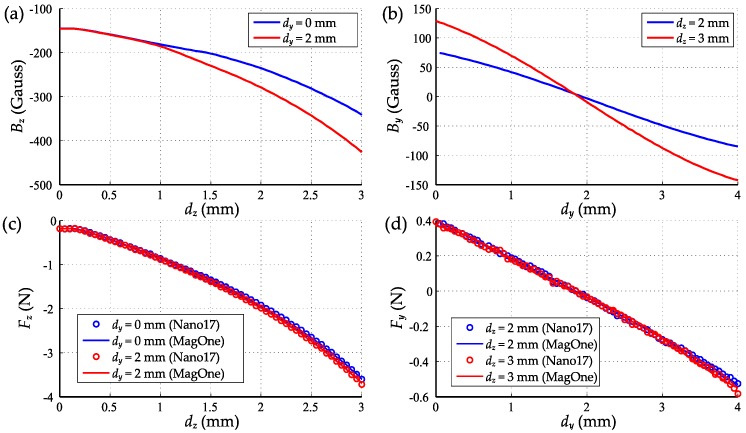
(**a**) *B_z_* during the *z* axis indentation (applying normal force) with different shear forces applied; (**b**) Normal force output during the *z* axis indentation with different shear forces applied (the circle represents the reference force from Nano17; the line represents the calibrated output from MagOne); (**c**) B_y_ during the *y* axis indentation (applying shear force) with different normal forces applied; (**d**) Shear force output during the y axis indentation with different normal forces applied (same configuration as (**b**)).

The sensors were calibrated using the 2D scanning process shown in Figure 10 with a range of 3 mm in the *z* axis, 4 mm in the *y* axis and a step size of 50 μm. The resultant dataset was processed with the MLS method to determine the coefficients in Equation (11) with a polynomial order of four (n = 4), enabling the three-axis force vector to be calculated directly from the three magnetic field signals. Figure 11 shows the comparison of the calibrated force output from MagOne and the reference force from the F/T sensor Nano17 during the 2D scanning process. The RMS errors of the regression equation are 7.07 mN and 7.78 mN for shear force and normal force. Using a polynomial order over four would not further reduce the error, as the resolution of the F/T sensor is 6.5 mN. Figure 12a shows the magnetic field *B_z_* during the *z* axis indentation with different shear forces applied (tangential indentation *d_y_*), which shows a strong crosstalk effect (*B_z_* at *d_y_* = 0 mm is 30% larger *d_y_* = 2 mm when *d_z_* = 3 mm). Figure 12b shows the same effect in the *y* axis, demonstrating that as the *z* axis indentation increases, so does the sensitivity of *B_y_* to *d_y_*. Figure 12c,d shows that the calibrated output of the sensor has close agreement with the reference measure across load conditions, meaning that crosstalk effects between axes are eliminated.

### 4.2. Performance Evaluation and Demonstration

**Figure 13:**
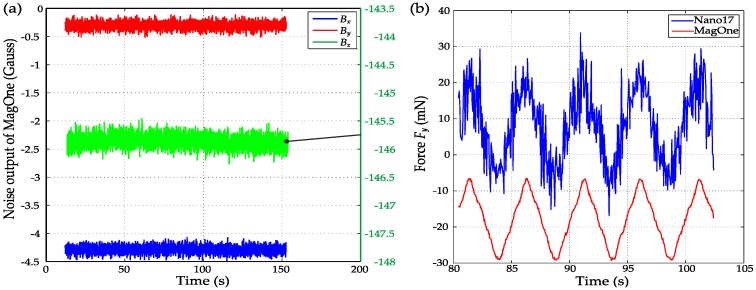
(**a**) Noise of the MagOne output (unloaded); (**b**) Shear force measurement comparison of MagOne and Nano17.

According to the results shown in Figure 12, the sensitivity of MagOne for force measurement is between 42~83 Gauss/N in the *z* axis and 85~298 Gauss/N in the *x*/*y* axis. As shown in Figure 13a, the RMS noise of the magnetic field in our environment is 0.11 Gauss in the *z* axis and 0.06 Gauss in the x/y axis. From Equation (11), the resolution range of the MagOne sensor was calculated as 1.42 mN to 0.72 mN in the *z* axis and 0.71 mN to 0.23 mN in the *x*/*y* axis. Considering that the resolution of the sensor will vary with the operating conditions, we use the worst-case resolution (1.42 mN in normal force and 0.71 mN in the shear force) to evaluate the performance of the sensor. When operating within the calibrated range, the maximum normal force is approximately 3.4 N; thus, the resolution can be described as 0.04% of full scale (FS). Similarly, in the shear force measurement, the resolution is 0.71 mN or 0.07% of full scale. To test the shear force measurement resolution of MagOne, a 3.3 N normal force (*z* axis) was applied, then the sensor tip was displaced with a 0.1-mm triangle sweep in the *y* axis. The output from MagOne is shown in Figure 13b with a shear force amplitude of approximately 22 mN, closely matching the reference sensor, but with significantly improved resolution and noise characteristics.

The calculations above consider a scenario in which the noise induced from external magnetic fields (both geomagnetic and from the local environment) is static and invariable with respect to the sensor. However, in many applications, this may not be the case, and the environmental noise will vary during operation. These factors are difficult to quantify because they are particular to the application; here, we consider an illustrative example of a sensor mounted on the manipulator of a small robotic arm actuated by DC electric motors. Rotation of the arm will change the orientation of the sensor with respect to the non-symmetric geomagnetic field and thus induce measurement error. A rotation of 180° will invert the influence of the static magnetic field; thus, for a 0.4 Gauss geomagnetic field, an error of up to 20 mN (approximately 0.6% FS) could be induced. In addition, the DC motors on the robotic arm will induce localised magnetic fields. To investigate this effect, a DC motor (A-max EC 32 Ø32 mm, Maxon, Sachseln, Switzerland) was operated at constant voltage (12 V) and fixed distances (30–180 mm in 50-mm steps) along the x axis from the MagOne sensor, and the influence of the motor location was determined. At 30 mm, the magnetic field from the motor induced a maximum error of approximately 10 mN in the measured normal force. This effect decayed rapidly with distance, showing 2 mN at 80 mm and a negligible effect thereafter. Thus, during the design of the robotic manipulator, it would be advantageous to monitor the sensor orientation and to locate drive motors an appropriate distance from the tactile sensor to minimise the influence from external magnetic noise. Once the sensor is designed, its sensitivity can be determined, and since the resolution of the sensor is dependent on the environmental noise, the lower the noise, the better the resolution and vice versa.

**Figure 14:**
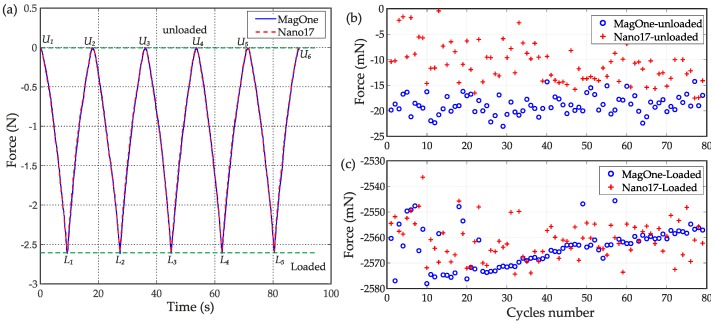
(**a**) Five cycles of the indentation test result; (**b**) Unloaded force output of MagOne and Nano17 for 80 cycles of indentation test; (**c**) Loaded force output (same as (**b**)).

To further investigate the performance of MagOne, tests were undertaken to explore the repeatability and stability characteristics. Firstly, the sensor was indented in the *z* axis repeatedly with a displacement range of 0–2.5 mm. Figure 14a shows the resultant force output (*F_z_*) from both MagOne and Nano17 during five cycles of the indentation, which demonstrates that the output of the MagOne sensor matches closely the reference force throughout the range. When the sensor was fully loaded (*d_z_* = 2.5 mm), the resultant force is approximately 2.56 N. Figure 14b,c show the unloaded (*d_z_* = 0 mm, points *U_i_* in Figure 14a) and loaded (*d_z_* = 2.5 mm, points *L_i_* in Figure 14a) force output of MagOne and Nano17 over 80 cycles. The results show that the repeatability of MagOne (1.8 mN, standard deviation) is better than Nano17 (3.8 mN standard deviation) when they are unloaded; while when they are loaded, MagOne and Nano17 have similar output repeatability (7.7 mN and 7.5 mN standard deviation, respectively). Furthermore, the figures show that the MagOne sensor has comparable stability to Nano17 during the 23 min of the indentation test (80 cycles). 

**Figure 15:**
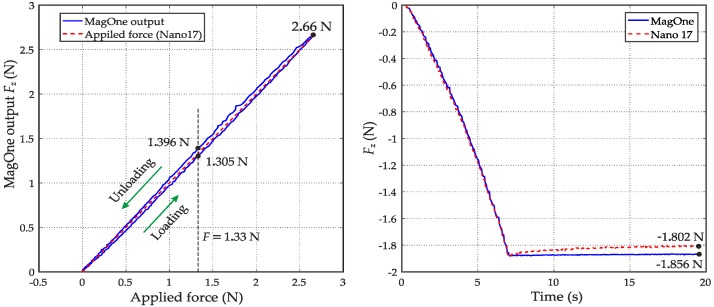
(**a**) Hysteresis error of the MagOne sensor; (**b**) Viscoelastic creep effects in the MagOne elastomer.

As discussed in Section 2.3, hysteresis and creep are very common issues for soft tactile sensors because of the viscoelastic behaviour of the elastomer material. The output of the MagOne sensor during a cycle of loading and unloading is plotted in Figure 15a, which shows a maximum hysteresis error of 3.4% (0.091 N) of the maximum applied force (2.66 N). To test the influence from creep effects in the silicone material, the MagOne sensor was indented by 2 mm and held in this deformed state for an extended period. Figure 15b shows that the output of MagOne sensor is −1.856 N at the end of the indentation and maintains the same value because the magnet is stationary with respect to the Hall effect sensor. The force output from the Nano17 sensor reaches a similar maximum then slowly drifts back to −1.802 N after 15 s because of the creep effects in the MagOne elastomer. These hysteresis creep effects are minimised in the MagOne sensor because the magnet is close to the top surface of the elastomer, as discussed in Section 2.3.

**Figure 16:**
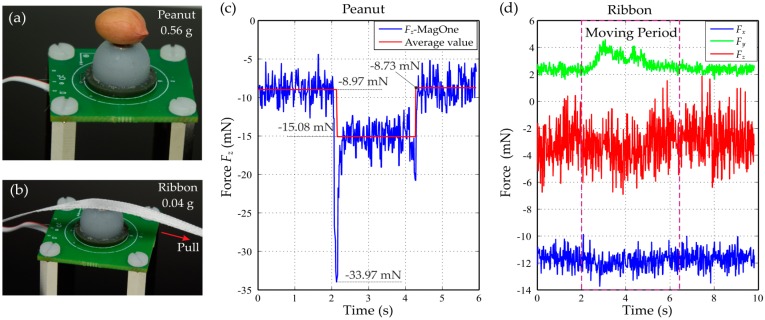
Demonstrations: (**a**) A photograph of the MagOne with a peanut on top; (**b**) A photograph of the MagOne with a ribbon; (**c**) Normal force output *F_z_* during drop down and pick up; (**d**) The three-axis force output when pulling a soft, thin ribbon across the top surface of MagOne along the *y* axis.

Two experiments (Figure 16a,b) were conducted to provide a tangible illustration of the capability of the MagOne sensor for normal and shear force measurement. Firstly, a peanut (0.56 g) was gently dropped down onto the top surface of the MagOne sensor, then picked up. Figure 16c shows the normal force *F_z_* response, which is sufficiently sensitive to register a 20 mN impact force as the peanut is dropped, then stabilising to show the weight of the peanut before returning quickly to the unloaded value when the peanut was picked up. Secondly, to examine shear force performance, a soft thin ribbon (5 mm wide, 80 mm long, 0.04 g) was slowly pulled across the top surface of the sensor along the y axis. As shown in Figure 16d, the resultant sensor output from MagOne shows clear increases in shear force during movement (caused by the friction of the ribbon against the silicone surface), which are as small as 1.5 mN.

## 5. Conclusions and Discussion

This manuscript presents a design methodology for soft tactile sensors based on 3D magnetic sensing technology to enable researchers from different disciplines to develop custom sensors for their own applications, as illustrated by our case study of the MagOne tactile sensor.

The analysis presented in Section 2 illustrates that while a magnetic field-based tactile sensor is conceptually simple, there are a wide range of design variables (e.g., size, compliance, measurement range and resolution) that can be manipulated in the pursuit of a system that satisfies a given set of performance requirements. The design guidelines here are derived from a detailed consideration of the underlying physical, electromagnetic and electronic aspects, which govern soft magnetic-based tactile sensors. Firstly, this enables a more efficient design process in which particular aspects of the sensor can be prioritised and their effect on other aspects understood, for example the compromise between sensor range and sensitivity for a given magnet size. Secondly, a detailed appreciation of the sensor’s characteristics enables the use of appropriate calibration methods to link the measured magnetic field strength with a corresponding force. A key consideration here is decoupling the axes such that the cross-talk effect in the calibrated force output is eliminated. 

In the case study, our approach exploits a modern commercially-available 3D Hall sensor chip and combines it with a cylindrical magnet and cast silicone elastomer, providing a system that is soft, low-cost, easy to fabricate and robust. The design and calibration process is highlighted in the development and evaluation of our MagOne sensor. This exemplifies the use of low-cost technology to form a high precision tactile sensor. The MagOne prototype costs only a few dollars, but it is capable of providing accurate three-axis force measurement with a higher resolution than the commercial F/T sensor (Nano17) used in our calibration process (see Section 3.3). 

The form of magnetic field-based soft tactile sensor presented here has many virtues, with key attributes, including low-cost, durability, mechanical compliance, measurement sensitivity and accuracy, robustness to environmental contaminants (liquid, dust, non-magnetic medium, etc.) and a form factor that is convenient for integration into other applications. However, the current approach does have some limitations that should be acknowledged;

- Tilt effect: The magnetic field will also be changed when the magnet is tilted, which will currently be interpreted as a linear movement and, thus, introduce error into the force measurement. By using a second two degree of freedom reference sensor alongside the main 3D magnetic sensor, the tilt effect can be detected and compensated. Alternatively, the elastomer structure could be specifically designed to minimise tilt movement of the magnet and, thus, the induced error, if this were a particular requirement of the application.
- Environmental interference: The variation of the magnetic field from other sources in the environment will influence the sensor measurement if their variation exceeds the resolution of the sensor. Since magnetic field strength decays rapidly with distance, these disturbances are negligible in many environments or can be minimised through careful design (e.g., using shielding or locating localised magnetic sources at a distance from the sensor). Using a second sensor as a reference would also help to minimise the noise from an external magnetic source, as discussed below.
- Interaction with ferromagnetic material: An inherent shortcoming of this technology is that the sensor cannot be used to interact with objects composed of ferromagnetic materials, since these will significantly alter the magnetic field source and, thus, induce large measurement errors.

We have identified two particular areas for future development of this research. Firstly, the analyses and evaluation presented here provide a detailed investigation into the quasi-static operation of soft magnetic sensor systems. It is evident that these systems have much potential for dynamic measurement and that these aspects (e.g., dynamic response and bandwidth) must be fully investigated, documented and considered during design and operation. Secondly, the methods presented here scale to allow the consideration of multi-nodal systems. In this form factor, multiple Hall sensors and magnetic sources are combined. This can be used to enhance:

- Measurement DoFs: monitoring multiple contact points or considering rotational aspects (e.g., the tilt effect discussed above), three-axis force mapping and objects’ topography reconstruction;
- Performance: measurement signals can be combined to improve measurement sensitivity and accuracy;
- Robustness: multiple measurement nodes enable the use of noise-suppression techniques to minimise background noise.

In summary, it is evident that magnetic field-based soft tactile sensors have significant potential. Their characteristics make them well suited for applications in emerging and growing areas of research, such as soft robotics and medical technologies. Equally, the underlying engineering science presents many opportunities for further work, as outlined above, that will help to develop and mature the technology, lowering technological barriers to bring more widespread adoption.

## Supplementary Materials

The followings are available online at http://www.mdpi.com/1424-8220/16/9/1356/s1, Video S1: MagOne demo: Finger tap & feather touch.

## Abbreviations

The following abbreviations are used in this manuscript:

MEMS	Microelectromechanical system
FEM	Finite element method
I2C	Inter-Integrated Circuit, alternatively written as I^2^C
SPI	Serial Peripheral Interface
RMS	Root mean square
MLS	Moving least square
PCB	Printed circuit board
F/T	Force/torque
FS	Full scale
DoFs	Degrees of freedom
